# Social inequalities in the participation and activity of children and adolescents with leukemia, brain tumors, and sarcomas (SUPATEEN): a protocol for a multicenter longitudinal prospective observational study

**DOI:** 10.1186/s12887-020-1943-3

**Published:** 2020-01-31

**Authors:** Julia Roick, Reinhard Berner, Toralf Bernig, Bernhard Erdlenbruch, Gabriele Escherich, Jörg Faber, Christoph Klein, Konrad Bochennek, Christian Kratz, Joachim Kühr, Alfred Längler, Holger N. Lode, Markus Metzler, Hermann Müller, Dirk Reinhardt, Axel Sauerbrey, Florian Schepper, Wolfram Scheurlen, Dominik Schneider, Georg Christof Schwabe, Matthias Richter

**Affiliations:** 1 Institute of Medical Sociology (IMS), Martin Luther University Halle-Wittenberg, Magdeburger Str. 8, 06112 Halle (Saale), Germany; 2 Department of Pediatrics, University Hospital Carl Gustav Carus, Technische Universität Dresden, Dresden, Germany; 3 Department of Pediatrics, Martin Luther University Halle-Wittenberg, Halle, Germany; 4 Johannes Wesling Klinikum Minden, University Hospital for Children and Adolescents, Ruhr University Hospital, Bochum, Germany; 5 Clinic of Pediatric Hematology and Oncology, University Medical Center Hamburg-Eppendorf, Hamburg, Germany; 6 Children’s Hospital, Pediatric Hematology, Oncology and Hemostaseology, University Medical Center of the Johannes Gutenberg University Mainz, Mainz, Germany; 7 Department of Pediatrics, Dr. von Hauner Children’s Hospital, University Hospital, Ludwig-Maximilians-University Munich, Munich, Germany; 8 Department of Pediatric Hematology and Oncology, University Hospital, Frankfurt/Main, Frankfurt, Germany; 9 Department of Pediatric Hematology and Oncology, Hannover Medical School, Hannover, Germany; 10 Clinic for Children and Adolescent Medicine, Klinikum Karlsruhe, Karlsruhe, Germany; 11 Department of Integrative Pediatric and Adolescent Medicine, Gemeinschaftskrankenhaus Herdecke, Herdecke, Professorship for Integrative Pediatrics, Center for integrative medicine, Faculty of Health, University of Witten/Herdecke, Witten, Germany; 12 Department of Pediatrics, Children’s University Hospital, University of Greifswald, Greifswald, Germany; 13 Department of Pediatrics and Adolescent Medicine, University Hospital Erlangen, Erlangen, Germany; 14 Department of Pediatrics and Pediatric Hematology/Oncology, University Children’s Hospital, Klinikum Oldenburg AöR, Carl von Ossietzky University, Oldenburg, Germany; 15 Pediatric Hematology and Oncology, University of Duisburg-Essen, Essen, Germany; 16 Clinic for Children and Adolescent Medicine, Helios Klinikum Erfurt, Erfurt, Germany; 17 Department of Pediatric Oncology, Hematology and Hemostaseology, Leipzig University, Leipzig, Germany; 18 Cnopf’sche Children Clinic, Clinic Hallerwiese, Nürnberg, Germany; 19 Clinic for Children and Adolescent Medicine, Klinikum Dortmund, Dortmund, Germany; 20 Children’s Hospital, Carl-Thiem-Klinikum, Cottbus, Germany

**Keywords:** Children and adolescents, Cancer, Social participation, Patient reported outcomes, Brain tumors, Leukemia, Sarcomas

## Abstract

**Background:**

About 2000 children and adolescents under the age of 18 are diagnosed with cancer each year in Germany. Because of current medical treatment methods, a high survival rate can be reached for many types of the disease. Nevertheless, patients face a number of long-term effects related to the treatment. As a result, physical and psychological consequences have increasingly become the focus of research in recent years. Social dimensions of health have received little attention in health services research in oncology so far. Yet, there are no robust results that allow an estimation of whether and to what extent the disease and treatment impair the participation of children and adolescents and which factors mediate this effect. Social participation is of great importance especially because interactions with peers and experiences in different areas of life are essential for the development of children and adolescents.

**Methods:**

Data are collected in a longitudinal, prospective, observational multicenter study. For this purpose, all patients and their parents who are being treated for cancer in one of the participating clinics throughout Germany will be interviewed within the first month after diagnosis (t1), after completion of intensive treatment (t2) and half a year after the end of intensive treatment (t3) using standardized questionnaires. Analysis will be done by descriptive and multivariate methods.

**Discussion:**

The results can be used to identify children and adolescents in high-risk situations at an early stage in order to be able to initiate interventions tailored to the needs. Such tailored interventions will finally reduce the risk of impairments in the participation of children and adolescents and increase quality of life.

**Trial registration:**

ClinicalTrials.gov: NCT04101123.

## Background

In Germany, approximately 2000 children and adolescents under the age of 18 are diagnosed with cancer each year [1]. Leukemias are the most common malignancies in children and adolescents, accounting for approximately 33% of all cancers, followed by brain tumors (25%). Other common malignancies in childhood are soft tissue sarcomas (6%) and bone tumors (5%) [1, 2]. Because of more differentiated diagnostics and standardized treatment protocols, survival rates have increased significantly in the last several decades [3]. Across all cancers, the 5-year survival rate is 85%. The probability of living more than 10 years after diagnosis is only slightly below this value [4]. The prognosis of brain tumors and sarcomas is worse than that of leukemias and highly depends on localization, tumor size, pathology, and possibilities of tumor removal [2].

Due to this success of treatment, cancer in childhood has changed from an acute life-threatening to a curable illness. However, the price for this cure often lies in a not inconsiderable rate of long-term consequences to which not much is known yet. Thus, late effects addressed by patients have gained attention in pediatric oncology research. In addition to the consequences of chemotherapy and radiation (e.g., fatigue, emotional distress), other effects may develop later, such as fertility disorders, metabolic disorders, secondary malignant tumors, cognitive impairments, and cardiac problems [5–7]. Some of these conditions even develop years after the end of treatment. Even young adults who have been treated for cancer in childhood still report neurocognitive impairments and reduced vitality and suffer from sleep disturbances and fatigue [8]. These issues require an adaption of one’s lifestyle, for example, a reduced workload with respect to school, work, or even leisure activities [9, 10]. The effects for children and adolescents are particularly serious, as they undergo important developmental phases during cancer treatment. Children’s ability to participate in social activities can be considerably limited [11]. However, interacting with peers is a fundamental component of children’s and adolescents’ development of social skills and competencies [12]. To avoid disadvantages in psychosocial development, it is important to quickly reintegrate children and adolescents with cancer into social life. There are already a number of studies that focus on the impact of childhood cancer on quality of life [13, 14]. Social dimensions of health, such as activity and participation, have rarely been investigated in pediatric oncology. Here, activity means the concrete execution of an action and participation the involvement in life situations. To date, no reliable results, neither international findings nor those for Germany, are available for use in estimating whether and to what extent the disease and treatment are associated with restrictions in social participation and the factors that mediate this effect. Furthermore, it is unclear to what extent social inequalities contribute to better or worse disease management by influencing personal and social factors.

### Study objectives

The aim of the study is to investigate the influence of social determinants, particularly the socioeconomic position of the parents, on participation and activity in children and adolescents between 10 and 18 years with leukemia, brain tumors and sarcomas. Furthermore, personal and treatment-related factors and their effects on participation will be explored.

**Fig. 1:**
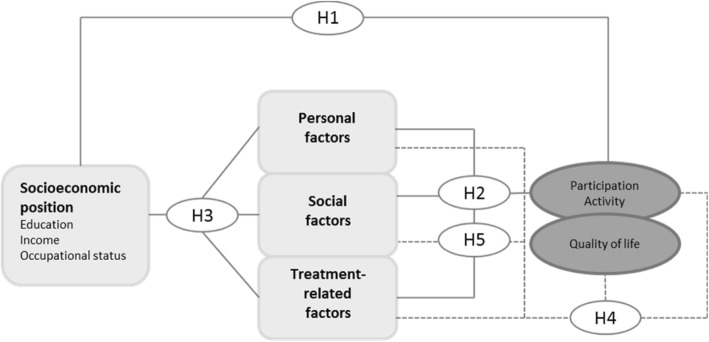
Study hypotheses

Our study hypotheses are as follows (Fig. 1):

1. Participation and activities in children and adolescents with leukemia, brain tumors and sarcomas vary during and after cancer treatment in relation to the socioeconomic position of the parents.
2. Personal, social, and treatment-related factors are associated with the participation and activity of children and adolescents with leukemia, brain tumors and sarcomas during and after cancer treatment.
3. The socioeconomic position of the parents influences personal, social, and treatment-related factors of children and adolescents with leukemia, brain tumors and sarcomas during and after cancer treatment and may therefore explain the findings for participation and activity.
4. Personal, social, and treatment-related factors as well as participation and activity are related to quality of life in children and adolescents with leukemia, brain tumors and sarcomas during different phases of cancer therapy and after treatment.
5. Predictors can be identified from personal, social, and treatment-related factors that can already be used to estimate a risk of low participation during cancer treatment.

Primary endpoints are social participation, activity, and quality of life. Secondary endpoints are illness perceptions, self-concept, self-efficacy, sense of coherence, social support, coping, optimism, psychosocial problems and strengths, mental health, fatigue, psychosocial needs and evaluation of the treatment.

## Methods/design

The study protocol complies with the STROBE guidelines for the reporting of observational studies.

### Study design

The SUPATEEN study is designed as a multicenter, longitudinal, prospective, observational study in Germany. The patients and one of their parents will be interviewed during the first month after diagnosis (t1), at the end of intensive treatment (t2), and half a year after end of intensive treatment (t3). At t1, paper-based data collection is conducted in the hospital. At t2 and t3, participants will have the choice to receive the questionnaire via mail or complete it online. The equivalence of written and electronic data collection is well-documented [15]. For the internet-based survey, the software CHES (Computer-Based Health Evaluation System) will be used. The software has already proven itself in practice and is supported by the European Organisation for Research and Treatment of Cancer (EORTC) [16].

### Eligibility

Patients aged 10–18 years of both sexes who are newly diagnosed with confirmed leukemia, brain tumors or sarcomas of all stages of cancer are eligible for this study. They must be admitted for the treatment of their disease in one of the participating study centers. Additionally, we include one parent of each patient. Parents must give written consent for the participation of their children if they are under the age of 18. The exclusion criteria are as follows: a) having a relapse or secondary tumor, b) insufficient command of German, c) profound cognitive and physical impairments, and d) no written informed consent.

### Recruitment

Any patient meeting the inclusion criteria will be informed about the study within the first month after diagnosis by the responsible clinic staff at the study center. After explaining the content and objectives of the study as well as the voluntary nature of participation and adherence to the protection of data of children and adolescents and their parents, the clinic staff will distribute the questionnaires. Completed questionnaires are returned to the clinic staff in a sealed envelope. Patient enrollment will last for 18 months. Reminders, questionnaires, and login details for follow-ups will be sent from the study center in Halle. In cases of refusal, patients are asked to provide the following information: their age, gender, disease type and reasons for declining participation for responder analyses.

### Sample size

The inclusion of many study centers and a large study population (encompassing patients with the three most common cancers in childhood and adolescence) facilitate the recruitment of an adequately sized sample. The participating clinics treat approximately 470 children and adolescents between the ages of 10 to 18 years with leukemia, brain tumors, and sarcomas per year. With a recruitment time of one and a half years, approximately 700 patients are eligible for the study. A conservatively estimated response rate of 70% [17] and a further 30% loss to follow-up [18] will leave approximately 340 complete cases. With this sample size, we can test our hypotheses and include a total of 30 independent variables and confounders [19].

### Instruments

Data collected during each survey time point are detailed below and in the Table 1.

**Table 1:** Measures and time points for patients and parents

Outcomes	Instruments	t1		t2		t3	
		Patient	Parent	Patient	Parent	Patient	Parent
Demographics	Standard inventory	x	x				
Clinical data	CRF	x		x		x	
Participation	CASP	x		x		x	
Evaluation of the treatment	FBB			x	x		
Self-concept	SDQ	x		x		x	
Fatigue	EORTC QLQ-C30	x		x		x	
Social Support	SSS	x		x		x	
Illness perception	IPQ	x		x		x	
Self-efficacy	SWE	x		x		x	
Optimism	BFW	x		x		x	
Autonomy	Kidscreen	x		x		x	
Psychosocial problems/strengths	SDQ-D	x		x		x	
Familial burden	FaBel				x		x
Family resources	FES		x				x
Psychosocial needs	SCNS-SF-34		x		x		
Satisfaction with life	SWLS		x		x		x
Doctor-parent relationship	PRA-D				x		
Quality of life	KINDL-R	x		x		x	
	SF-12		x		x		x
Sense of coherence	C-SOC	x		x		x	
	SOC-L9		x		x		x
Coping	CODI	x		x		x	
	CHIP-D		x		x		x
Mental health	ChilD-S	x		x		x	
	HADS		x		x		x

#### Sociodemographic and clinical data

Sociodemographic characteristics will be assessed via self-report with a standardized inventory. Clinical data will be ascertained from the medical records and include information about ICD-10 diagnosis, disease stage, current treatment, and comorbid conditions.

#### Social participation and activity

To evaluate social participation and activity, we will use the Child and Adolescent Scale of Participation (CASP) [20, 21]. This self-report questionnaire measures the extent to which children and adolescents participate in home, school, and community activities in comparison to their peers. The instrument consists of 20 items that form 4 subscales (home participation, school participation, community participation, home and community living activities).

#### Evaluation of the treatment

The Questionnaires of the Evaluation of Treatment (FBB) evaluate the therapy and quality of treatment from the children’s and parents’ perspectives [22]. The children’s and parents’ version consists of 20 items that can be summarized in a total score or scores on 3 subscales (success of the treatment, relationship to medical team, treatment conditions).

#### Self-concept

To assess self-concept, we use 3 dimensions (physical appearance, parent relations, peers) from the short version of the Self-Description Questionnaire (SDQ) [23, 24]. The questionnaire measures the self-concept of children in different domains via self-report. Every subscale consists of 3 items.

#### Fatigue

We will use the fatigue scale from the European Organization for Research and Treatment of Cancer Quality of Life Questionnaire (EORTC QLQ-C30) [25] to ascertain patients’fatigue symptoms.

#### Social support

Social support will be measured with the Social Support Scale (SSS) [26, 27]. The 8-item self-report instrument assesses support in terms of showing affection, listening, providing information, and engaging in activities together via self-report.

#### Illness perception

The revised version of the Illness Perception Questionnaire (IPQ-R) measures individual beliefs and feelings about an illness and is based on Leventhal’s self-regulatory model [28]. We will use the following 3 scales: personal control, treatment control, and coherence.

#### Self-efficacy

The generalized self-efficacy scale (GSE) consists of 10 items and assesses self-beliefs about coping with difficult demands in life (perceived self-efficacy) [29].

#### Optimism

We will use the 8-item scale of a positive attitude toward life from the Bern Subjective Well-Being Questionnaire for Adolescents (BFW) to evaluate optimism [30]. This scale ascertains a generally positive attitude (“see the good side”) as well as one’s personal conviction to lead a good life.

#### Autonomy

To evaluate children’s and adolescents’ autonomy, the 5-item autonomy scale from the Kidscreen questionnaire will be used [31]. This scale measures the opportunities to create social and leisure time and will be summarized in a total score.

#### Psychosocial problems and strengths

To assess psychosocial problems and strengths, we will use the Strengths and Difficulties Questionnaire (SDQ) for the self-report of children and adolescents [32]. The instrument consists of 25 items equally divided across 5 scales (emotional problems, conduct problems, peer problems, hyperactivity, and prosocial behavior).

#### Familial burden

Familial burden will be ascertained with the short form of the Impact on Family Scale (FaBel-11) [33]. The items are summarized in a total score and include questions about the general negative impact of parents, social relationships, and financial burden.

#### Family resources

A shortened version of the German adaption of the Family Environment Scale (FES) will be used to assess family resources [34, 35]. The instrument contains 12 items that form 3 subscales (cohesion, control, family activities) and a total score.

#### Psychosocial needs

To evaluate the psychosocial needs of the parents, the short form of the Supportive Care Needs Survey (SCNS-SF-34-D) will be used [36]. In this 34-item questionnaire, unmet needs will be reported in 5 domains (physical and daily living needs, psychological needs, patient care and support needs, health system and information needs, sexuality needs). For this study, the sexuality scale was omitted.

#### Satisfaction with life

The Satisfaction With Life scale is a 5-item instrument that measures global life satisfaction and subjective well-being [37]. Answers will be summarized in a total score.

#### Doctor-parent relationship

The quality of the doctor-parent relationship will be assessed with the Patient Reactions Assessment (PRA-D) [38, 39]. The instrument contains 15 items that are equally divided across 3 scales (information, affectivity, communication).

#### Quality of life

The KINDL questionnaire will be used to ascertain children’s and adolescents’ health-related quality of life [40]. Parents’ quality of life will be assessed with the 12-item Short Form Health Survey (SF-12) [41]. The instrument can be summarized in a total score for physical and mental health.

#### Sense of coherence

To assess sense of coherence in children and adolescents, the Children’s Sense of Coherence Scale (C-SOC) was used [42]. The instrument contains 12 items about children’s sense of comprehensibility, manageability, and meaningfulness, which will be summarized in a total score. Parent’s sense of coherence will be measured with a short form of Antonovsky’s Sense of Coherence Scale (SOC-L9) [43].

#### Coping

The coping strategies of children and adolescents were evaluated using the Coping with a Disease (CODI) questionnaire [44]. The 28 items of the instrument form 6 scales (acceptance, avoidance, cognitive-palliative, distance, emotional reaction, wishful thinking) and an overall rating of the disease management. The coping skills of parents will be assessed with the Coping Health Inventory for Parents (CHIP-D) [45]. This questionnaire measures parental coping with chronic childhood disease with 45 items that form 3 scales (maintaining family integration, maintaining social support, understanding health care situation).

#### Mental health

The Children’s Depression Screener (ChilD-S) will be used to assess depressive symptoms in children and adolescents [46]. The 8 items of the instrument are summarized in a total score. Parental mental health will be evaluated with the Hospital Anxiety and Depression Scale (HADS), which contains 14 items (7 per scale: anxiety, depression) [47].

### Statistical analysis

All collected data will be checked for consistency, validity, and missing values. Descriptive statistics will be calculated for all study variables, and their correlations will be explored. Therefore, the strength of the association between the different independent variables and participation can be examined. Differences in social participation and activity, depending on the socioeconomic position of the parents, will be conducted using multiple linear regression and variance analyses. Both will be controlled for confounders such as age, sex, stage of disease, disease site, and treatment-related factors. In addition, multiple regression analysis will also be applied to identify which personal, social, and treatment-related factors are associated with participation and activity and whether the socioeconomic position of the parents is associated with these intermediate factors. Depending on the frequency and type of missing data, listwise deletion of patients with missing data or appropriate imputation techniques will be applied.

### Bias control

To examine selection bias, we will compare responders and non-responders on sociodemographic (age and gender) and clinical (tumor stage and entity) characteristics. The loss to follow-up will be analyzed using more detailed personal and medical information. To keep missing responses to a minimum, patients with no response will receive questionnaires at most two times via mail and will be contacted by telephone and offered the opportunity to answer the questions in written form, online or by telephone. If the participant still declines further participation, he or she will be marked as an inactive participant, but the status can be changed back to active any time the participant changes his or her mind. Additionally, we will document the reasons for non-participation in the study.

### Ethical matters and data protection

The study is conducted in accordance with the Declaration of Helsinki. Ethical approval was obtained from the Ethics Committee of the Medical Faculty at Martin Luther University Halle-Wittenberg (reference number 2017–112) and from the Institutional Review Boards of each participating institute. Written informed consent will be obtained from all patients and their parents before enrollment.

All personal information is subject to professional discretion and data protection. Confidentiality is ensured by using pseudonyms (patient ID) with each questionnaire and case report form. Patient reported outcomes and clinical data will be stored separately from person-identifying information in a locker. The allocation list will be saved in a manner such that it is physically unlinked to the other data. The data will be accessible only to authorized study staff. The study has been registered at clinicaltrials.gov (NCT04101123).

## Discussion

For the first time, the study provides detailed results on the influence of social determinants on the social participation and activity of children and adolescents with leukemia, brain tumors, and sarcomas in Germany. Since these represent the three most common cancers in childhood and adolescents (> 60% of all new cases), meaningful insights can be gained for a large group of patients. Children and adolescents with impairments in social participation and quality of life can have health and emotional problems that should be recognized early. In addition, age-appropriate development can be hindered by limited interactions with peers. Thus, it is necessary to identify children and adolescents at risk of impaired social participation early in the course of treatment so that appropriate interventions can be initiated. In addition to the identification of risk groups, intermediary influencing factors are identified that can be used to explain inequalities in participation and activities.

## Trial status

The trial is ongoing. Patient enrolment has not yet started.

## Data Availability

The datasets used and/or analysed during the current study will be made available from the corresponding author on reasonable request.

## Abbreviations

T: time point
